# Enhanced refractive index sensitivity of localized surface plasmon resonance inflection points in single hollow gold nanospheres with inner cavity

**DOI:** 10.1038/s41598-022-11197-6

**Published:** 2022-04-28

**Authors:** Yun A Hong, Ji Won Ha

**Affiliations:** 1 Department of Chemistry, University of Ulsan, 93 Daehak-ro, Nam-gu, Ulsan, 44610 South Korea; 2 Energy Harvest-Storage Research Center (EHSRC), University of Ulsan, 93 Daehak-ro, Nam-gu, Ulsan, South Korea

**Keywords:** Sensors, Optical spectroscopy

## Abstract

Hollow gold nanoparticles have great potential for localized surface plasmon resonance (LSPR) sensing. In this study, we investigated the refractive index (RI) sensitivities of single hollow gold nanosphere (HAuNS) with thin Au shell and inner cavity and single solid gold nanosphere (AuNS) in media with different RIs. The HAuNS exhibited a remarkable improvement in the RI sensitivity than the AuNS of similar size. The increased RI sensitivity of HAuNSs was ascribed to plasmon coupling between the inner and outer surface of the Au nanoshell. We then investigated the homogeneous LSPR scattering inflection points (IFs) to better understand the RI sensitivity of single HAuNS. The LSPR IF at the long wavelength side exhibited a better RI sensitivity compared to the wavelength shift of its counterpart LSPR maximum peak. Furthermore, the single HAuNS showed a remarkable improvement in the RI sensitivity at the LSPR IFs when compared to the AuNS of similar size. Therefore, we provided a new insight into the effect of inner cavity of HAuNS on the RI sensitivity of homogeneous LSPR IFs for use in LSPR-based biosensors.

## Introduction

Plasmonic gold nanoparticles (AuNPs) exhibit distinct localized surface plasmon resonance (LSPR) properties. The peak position of LSPR is strongly influenced by the size, shape, and refractive index (RI) of the surrounding medium around the nanoparticles^1,2^. Therefore, LSPR has been utilized for the detection of interactions with molecules near nanoparticle surfaces. A LSPR-based biosensor observes noticeable changes in the LSPR peak wavelength when target molecules are attached on the AuNP surfaces^3,4^.

AuNPs with hollow structures have shown great potential for biosensing because of their characteristic optical properties that are caused by their thin Au shell and inner cavity^5,6^. The most apparent difference between solid and hollow AuNPs is the existence of an inner cavity. Recent studies have shown that hollow AuNPs possess higher RI sensitivities than solid AuNPs^6–10^. For example, Sun et al. presented increased LSPR sensitivity of hollow Au nanospheres (HAuNSs) compared with that of solid Au nanoparticles in response to environmental RI changes^10^. Wang et al. demonstrated a simple label-free, three-dimensional hierarchical plasmonic sensor that was based on HAuNSs^11^. Zhang et al. proposed an electrochemiluminescence biosensor using hollow AuNPs and graphene^12^.

Nevertheless, LSPR biosensors have been prone to the occurrence of asymmetric broadening in a LSPR peak while detecting variation in the local environment on nanoparticle surfaces^13^. The asymmetrical nature of a LSPR peak negatively affects the sensing efficiency^14^. Some studies stated that the aforementioned limitation can be significantly minimized by using the changes in the homogeneous LSPR inflection points (IFs) of AuNPs with respect to variations in local RI^15–17^. However, there have been limited studies on the elucidation of RI sensitivity in hollow AuNPs at single-particle level. Furthermore, the influence of the inner cavity of single hollow AuNPs on RI sensitivity of LSPR IFs has not been studied.

In the present study, we conducted single-particle studies to investigate the LSPR sensing effect of single gold nanospheres (AuNSs) and HAuNSs that were fixed on a glass slide with three different surrounding media, including air, water and oil with known RI values. In particular, we elucidated the RI sensitivity at LSPR IFs of the homogeneous scattering spectra obtained for solid AuNSs and HAuNSs with similar size.

## Results and discussion

**Figure 1:**
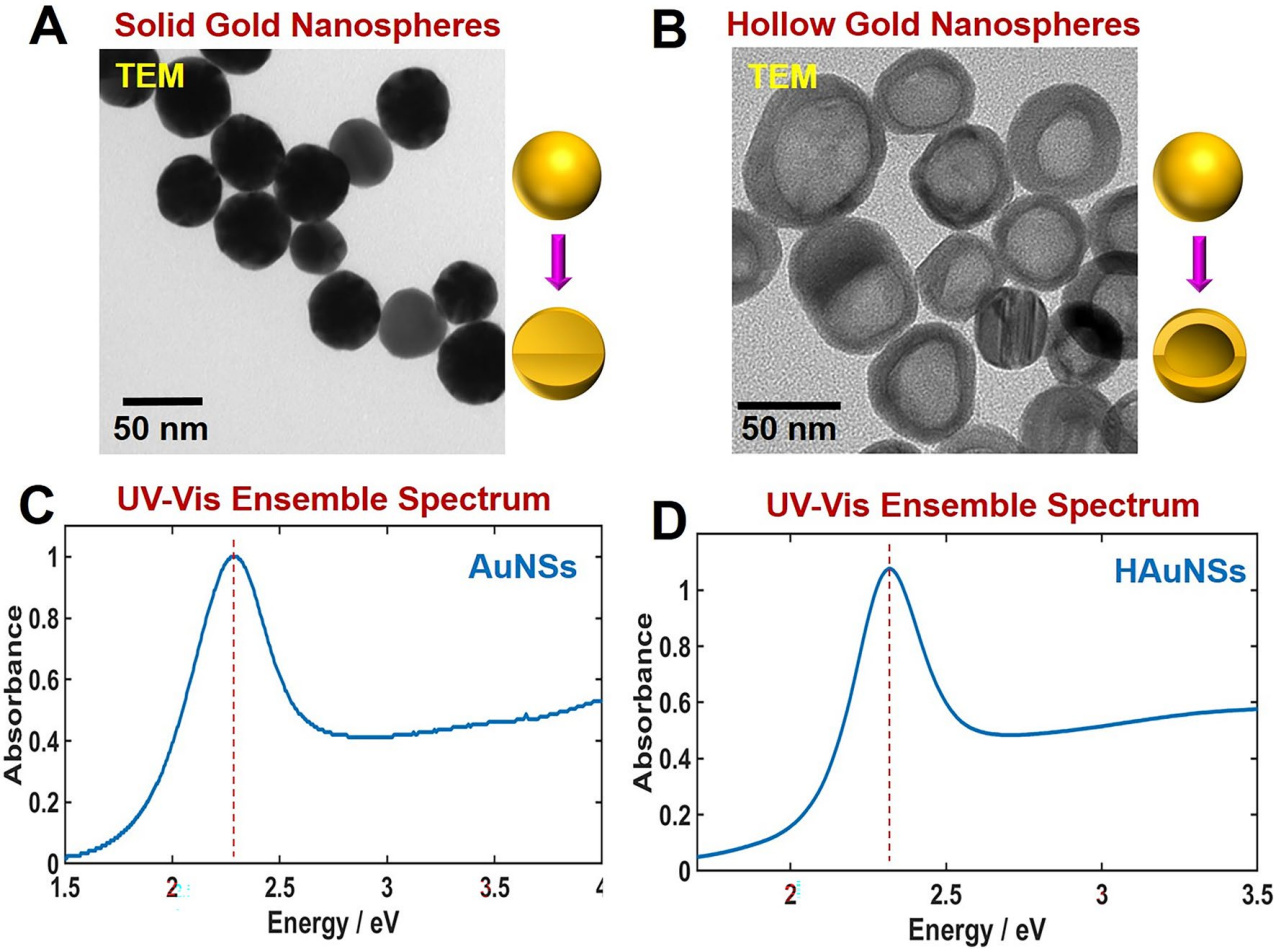
Representative SEM images of (**A**) AuNSs and (**B**) HAuNSs with inner cavity. UV–Vis extinction spectrum of (**C**) AuNSs and (**D**) HAuNSs dispersed in water.

The size and shape of AuNSs and HAuNSs were determined by transmission electron microscopy (TEM) and scanning electron microscopy (SEM). The TEM images of AuNSs and HAuNSs with inner cavity are shown Fig. 1A,B, respectively. The average sizes were 50.4 (± 2.69) nm and 53.9 (± 4.21) nm, respectively (Fig. S1). A TEM image showing multiple HAuNSs is provided in Fig. S2. We also provided a SEM image to show multiple HAuNSs in Fig. S3. The thickness of the Au shell of HAuNSs was approximately 10 nm (Fig. S4). The UV–Vis ensemble spectra of AuNSs and HAuNSs shown in Fig. 1C,D, respectively, were obtained with a Varian Cary 300 UV–Vis spectrophotometer. The LSPR peaks appeared at around 2.26 eV (548 nm) for both AuNSs and HAuNSs dispersed in water. However, single-particle measurements were necessary to clarify their optical property without the ensemble measurements that typically have heterogeneity issues.

**Figure 2:**
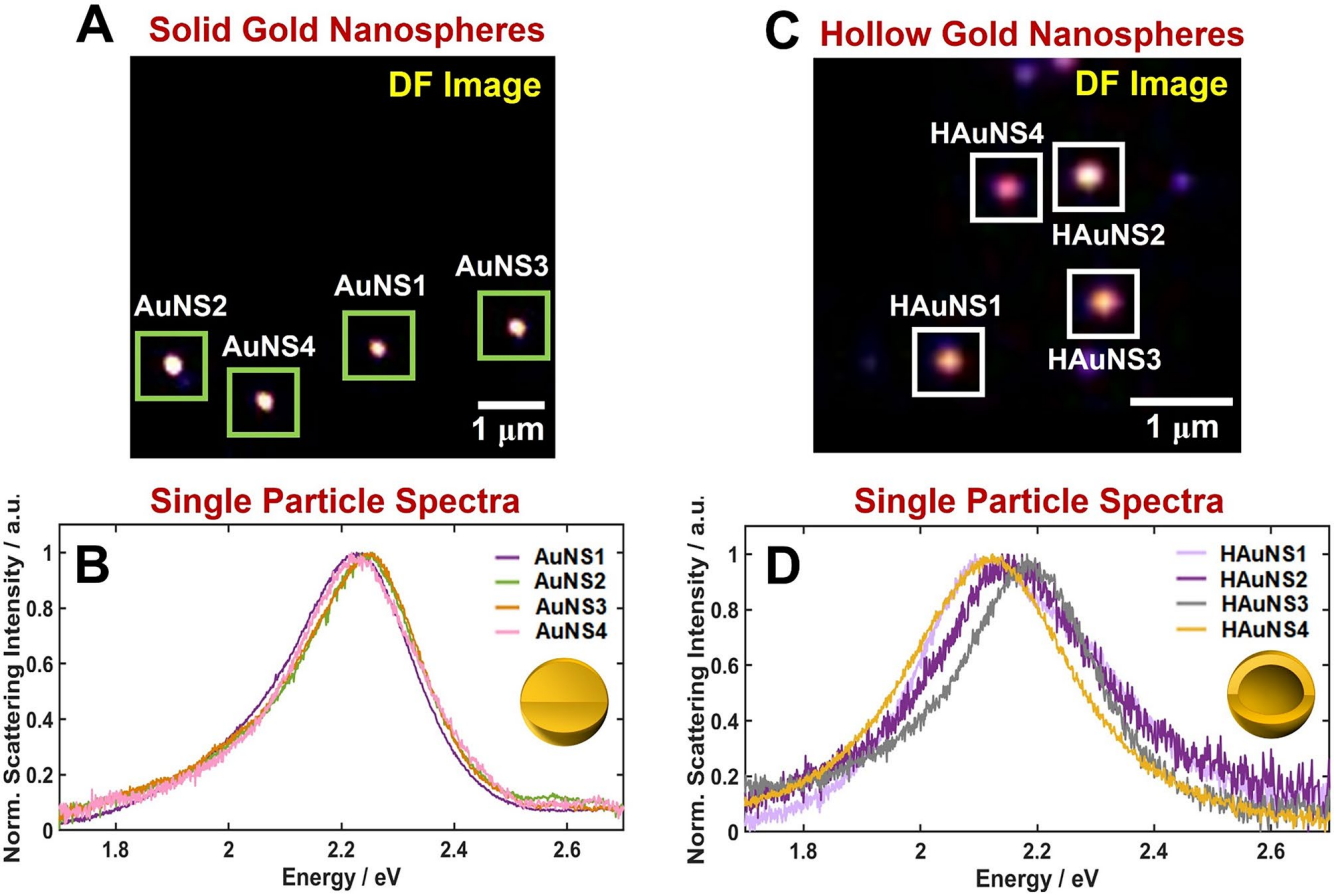
(**A**) Dark-field image of single AuNSs illuminated by white light. (**B**) Scattering spectra of the single AuNSs from the green square in (**A**). (**C**) Dark-field image of single HAuNSs illuminated by white light. (**D**) Scattering spectra of the single HAuNSs from the white square in (**C**).

In this study, we employed dark-field (DF) microscopy and spectroscopy to investigate the characteristic optical properties of AuNSs and HAuNSs at the single-particle level^18^. Figure S5 shows the experimental setup used for single-particle characterization using DF microscopy and spectroscopy. The AuNPs in aqueous solutions were drop casted onto a pre-cleaned glass slide for DF scattering measurements. The AuNPs were then illuminated by focusing a white light lamp through a high numerical aperture (NA) oil condenser. The scattered light from the nanoparticles was only sent to the objective lens in DF microscopy and spectroscopy (Fig. S6). A DF image of single AuNSs with an average size of 50.4 nm is shown in Fig. 2A. Moreover, the single-particle scattering spectra of four AuNSs which are highlighted with a green square in Fig. 2A are shown in Fig. 2B. The scattering spectra of single AuNSs in water showed a broad LSPR peak at approximately 2.23 eV (556 nm). The LSPR peak was further confirmed by the single-particle scattering spectra of multiple AuNSs in Fig. S7A. A DF scattering image of single HAuNSs with an average size of 53.9 nm is shown in Fig. 2C. The single HAuNSs also showed a broad LSPR peak at around 2.18 eV (568 nm) (Fig. 2D and Fig. S7B). We note that HAuNSs with inner cavity and AuNSs of similar sizes exhibited very similar single broad LSPR peaks in their scattering spectra. However, the scattering intensity of the HAuNSs was much smaller than that of the AuNSs (Fig. 2 and Fig. S7).

**Figure 3:**
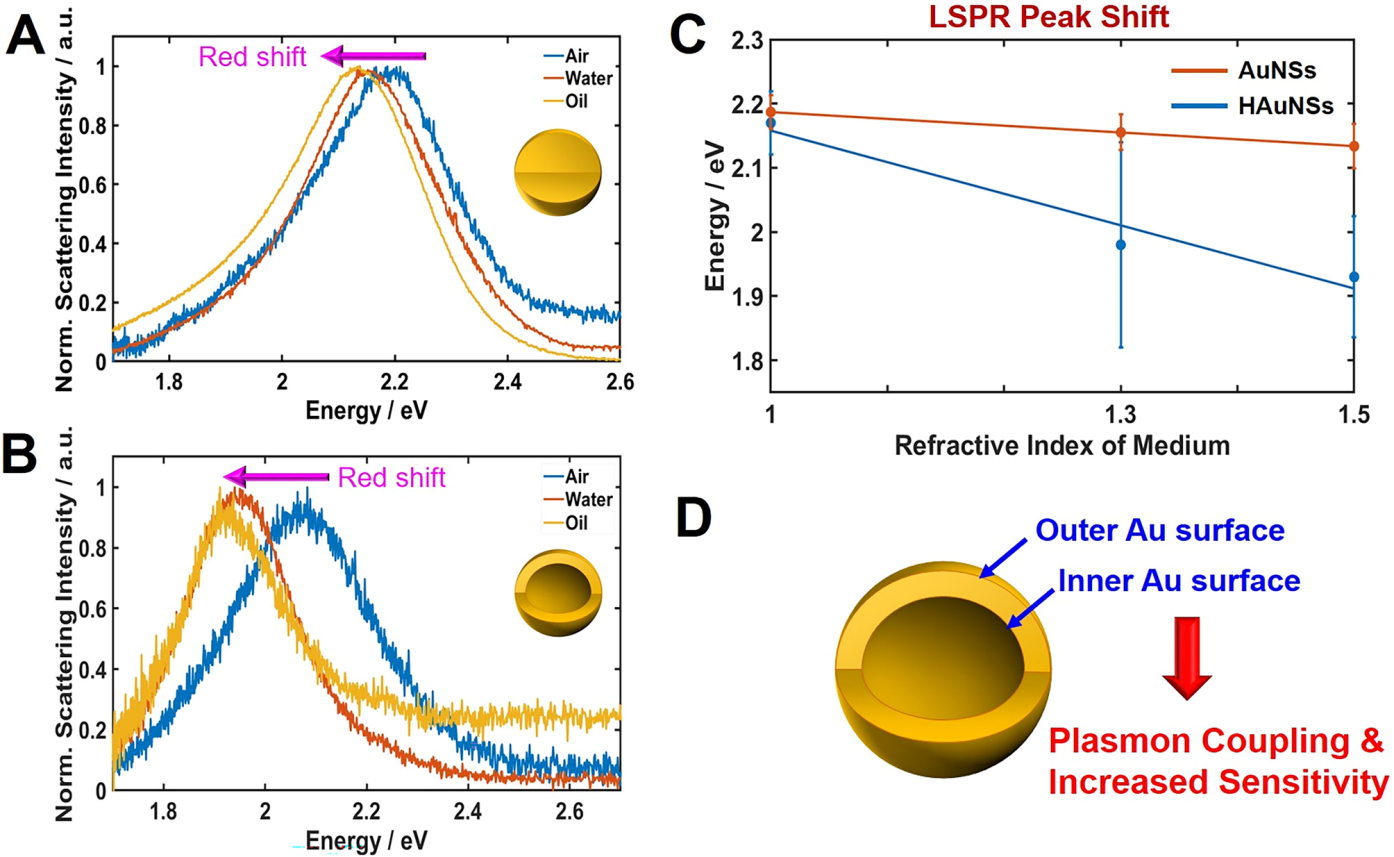
Change in the LSPR scattering spectra of single AuNS (**A**) and HAuNS (**B**) in the different local RI media: air, water, oil. (**C**) LSPR wavelength shifts for AuNS (red) and HAuNS (blue) as a function of the local RI of medium. (**D**) Schematic to show plasmonic coupling between the inner and outer surface of the Au nanoshell.

We first investigated the effects of varying the RI of a surrounding medium on the LSPR wavelengths of HAuNSs and solid AuNSs. To accomplish this, the scattering spectra of single AuNS and HAuNS were taken in three different RI media, including air, water and oil. The scattering spectra of a single AuNS and HAuNS that were fixed on a glass slide and surrounded by air, water or oil are shown in Fig. 3A,B, respectively. The LSPR wavelengths of both AuNS and HAuNS showed an increase when the RI values increased from air to oil, and this result was in good agreement with previous studies^1,16^. As shown in Fig. 3C, we compared the LSPR wavelength shifts as a function of the RI of surrounding medium for the AuNS and HAuNSs. Single HAuNSs with inner cavity showed higher LSPR wavelength shift (or slope) than the solid AuNSs of similar sizes. This indicated that the HAuNSs showed a higher RI sensitivity in the LSPR sensor, which was consistent with previous studies^5–7^. The increased RI sensitivity of HAuNSs having the Au shell thickness of ~ 10 nm can be mainly ascribed to the occurrence of plasmonic coupling between the inner and outer surface of the Au nanoshell (Fig. 3D). Recently, Shabaninezhad et al. investigated how shape, size, and aspect ratio affect the LSPR sensitivity in hollow Au nanostructures^19^. When decreasing the thickness of the shells, the plasmon coupling between the inner and outer surface of the Au shell increased, and the strong plasmon coupling resulted in the LSPR peak shift to longer wavelengths as well as the increase of its sensitivity^19,20^.

**Figure 4:**
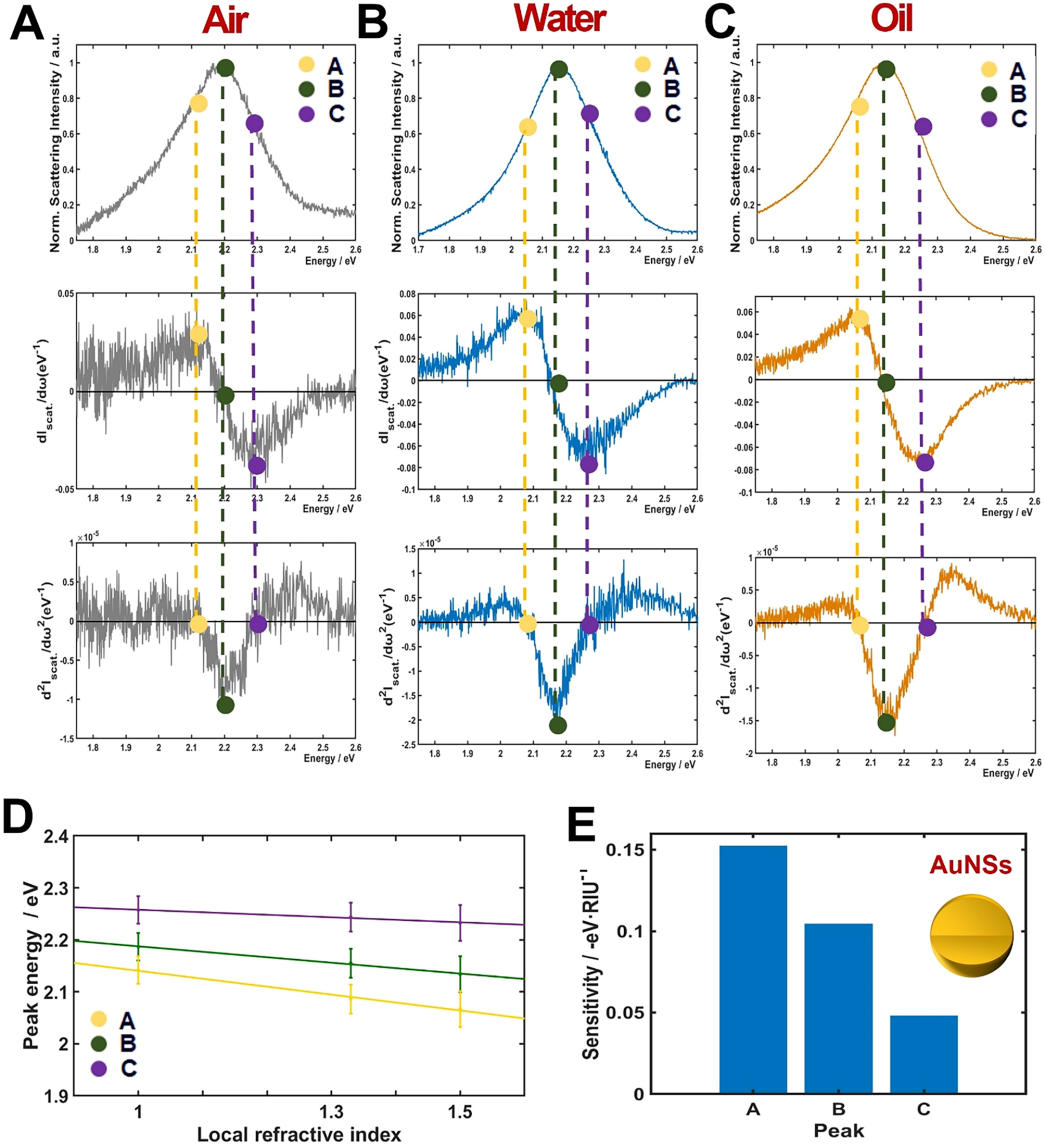
Inflection point method for single-particle LSPR scattering sensing with AuNSs in air, water and oil. (**A**–**C**) LSPR scattering efficiencies (first row) and their first (second row) and second (third row) order derivatives. (**D**) Peak energies plotted against the RI of air, water and oil for points A, B and C, respectively. (**E**) Sensitivity of local RI media on peak shifts A, B and C.

Recent studies showed that LSPR IFs have better RI sensitivities than a LSPR wavelength maximum peak of single AuNPs of various shapes^16,17,21,22^. Nevertheless, we have not found any study that presents the RI sensitivity at LSPR IFs of AuNPs with hollow structure. We therefore investigated the effect of inner cavity of HAuNSs on the RI sensitivity at LSPR IFs of homogeneous scattering spectra. The results were then compared with solid AuNSs of similar size. The scattering spectra of a single AuNS and their respective first- and second-order derivatives are shown in Fig. 4A–C (rows 1–3). The columns were distinguished by three different RI media used in this study (air, water, and oil). The LSPR peak maxima (represented by the legend B) in three different RI media occurred at 2.19, 2.16, and 2.13 eV, respectively. Furthermore, the local maxima and minima of the first-order derivatives (shown by markers A and C in Fig. 4A–C, respectively) occurred at 2.11/2.29, 2.08/2.26, and 2.04/2.24 (eV/eV) for air, water, and oil, respectively. We noted that the point B appeared at a value of 0 in the first-order derivatives of the LSPR scattering spectra (second row). In addition, the markers A and C representing the two LSPR IFs are observed at a value of 0 in the second-order derivatives of the LSPR scattering spectra (third row).

Then, we attempted to confirm the reproducibility of the experimental results shown in Fig. 4. We obtained the LSPR scattering spectra of more than 20 AuNSs in each local RI environment. The experimental data were in good agreement for all the AuNSs measured. The LSPR peak maxima (B) of 2.19 (± 0.036), 2.16 (± 0.038), and 2.14 (± 0.029) eV were obtained in air, water and oil, respectively. The values of LSPR IFs were 2.13 (± 0.073) (A) and 2.27 (± 0.062) eV (C) for air. The values were 1.94 (± 0.048) (A) and 2.24 (± 0.055) eV (C) for water. Finally, the values were 2.07 (± 0.056) eV (A) and 2.23 (± 0.057) eV (C) for oil. In the regime relevant to sensing, the peak energies showed linear functions with good approximation for three different RI environments^23^. We considered the peak energies in markers A, B, and C and evaluated their linearity in respect of the three different RI media used. The energy peaks for A, B, and C were plotted against the three different RI media (air, water, and oil) with corresponding values equal to 1.00, 1.33, and 1.52, respectively (Fig. 4D). A linear relationship was observed between the peak energies for A, B, and C and the local RI media. The slopes that were determined from a fitting function were 0.153 (R^2^ = 0.9398), 0.105 (R^2^ = 0.9983), and 0.048 eV·RIU^−1^ (R^2^ = 0.9895) for A, B, and C, respectively. It should be noted that the IF in A at longer wavelength sides exhibited a better sensitivity with regard to the IF in C at the shorter wavelengths side. The LSPR peak maxima (B) is shown in Fig. 4E. It should be noted that LSPR IF A at the longer wavelength side showed the highest sensitivity in regard to LSPR IF C (shorter wavelength side) and the LSPR maxima peak (B) as presented in Fig. 4E.

**Figure 5:**
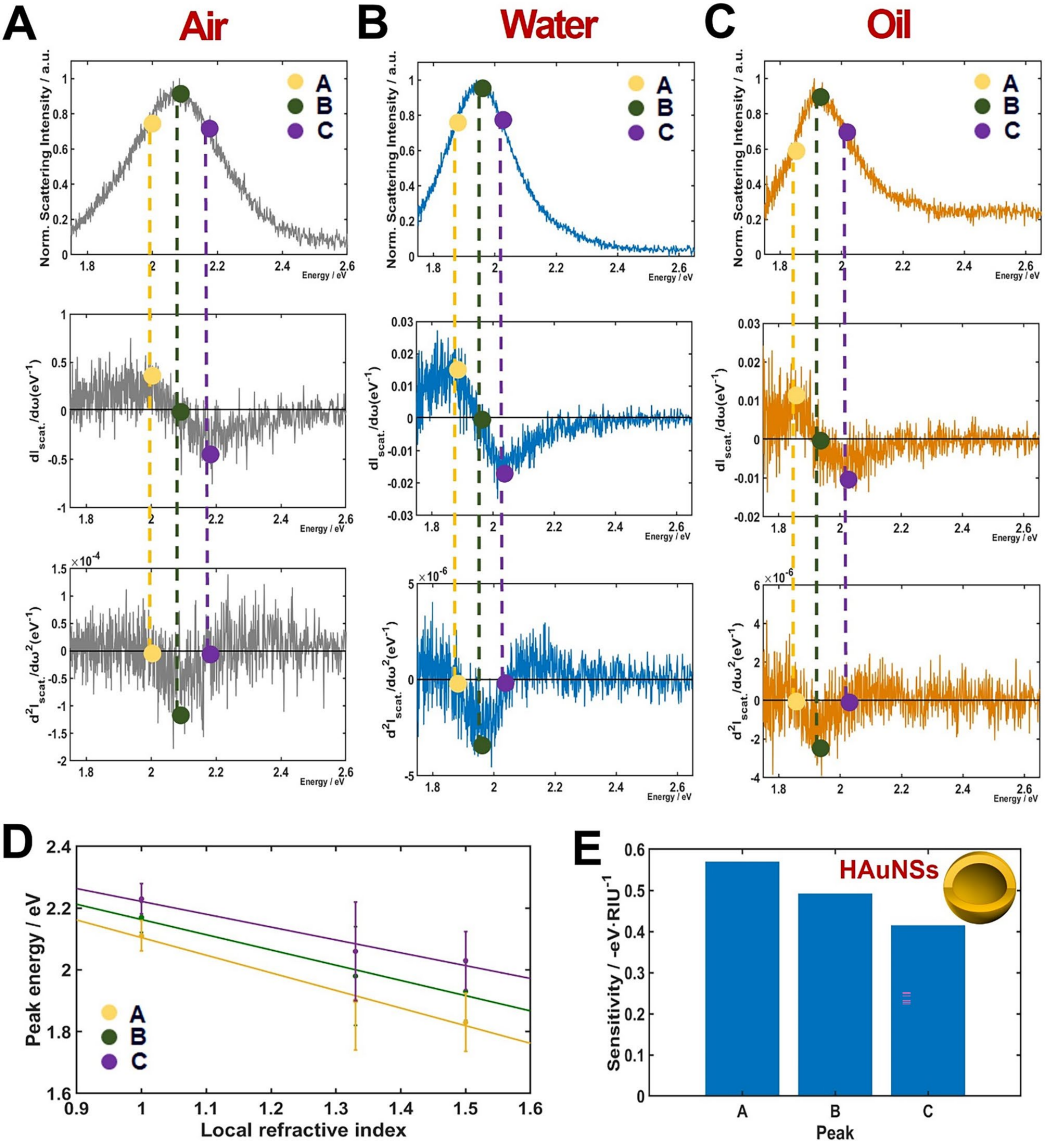
Inflection point method for the single-particle LSPR scattering sensing with HAuNSs in air, water and oil. (**A**–**C**) LSPR scattering efficiencies (first row) and their first (second row) and second (third row) order derivatives. (**D**) Peak energies plotted against the RI of air, water and oil for points A, B and C, respectively. (**E**) Sensitivity of local RI media on peak shifts A, B and C.

To deepen our understanding on the effect of inner cavity of HAuNSs on the RI sensitivity of the LSPR IFs, scattering experiments using DF microscopy and spectroscopy were carried out for HAuNSs. The RI sensitivity of HAuNSs at the LSPR IFs was compared with solid AuNSs with similar size as shown in Fig. 4. Similar to AuNSs in Fig. 4, the first- and second-derivatives of the LSPR scattering spectra of HAuNSs were also obtained. The experimental LSPR scattering spectra of a single HAuNS and its corresponding first- and second-order derivatives are shown in the first, second and third rows of Fig. 5A–C, respectively. The LSPR scattering peak maxima (B) were located at 2.09, 1.95 and 1.91 eV for air, water and oil, respectively. The local maxima and minima of the first-order derivatives (two LSPR IFs shown by markers in A and C, respectively) occurred at 1.99/2.19, 1.85/2.03 and 1.82/2.01 (eV/eV) for the three different RI media (air, water, and oil), respectively.

To check the compatibility of the experimental results shown in Fig. 5, we measured the LSPR scattering spectra of more than 20 HAuNSs in each local RI environment. The experimental data showed good agreement for all the HAuNSs analyzed. The LSPR peak maxima (B) were 2.08 (± 0.026), 1.98 (± 0.028), and 1.92 (± 0.037) eV for air, water and oil, respectively. Similarly, the LSPR IFs, A and C, were 1.98 (± 0.049) and 2.22 (± 0.049), 1.84 (± 0.160) and 2.05 (± 0.160), and 1.82 (± 0.094) and 2.02 (± 0.094) eV, for air, water, and oil, respectively. The peak energies for A, B, and C were plotted *vs.* local air, water, and oil RI media. A linear relationship was observed for the peak energies at A, B, C with respect to the three different local RI media, as presented in Fig. 5D. From the fitting function, the slopes were 0.570 (R^2^ = 0.9685), 0.493 (R^2^ = 0.9868) and 0.416 eV·RIU^−1^ (R^2^ = 0.9998) for A, B, and C, respectively. Similar to the results for the AuNSs, the LSPR IF A showed the highest sensitivity with regard to the LSPR IF C and the LSPR peaks maxima (B) as demonstrated in Fig. 5E. This result was consistent with the AuNSs (Fig. 4). Thus, a higher RI sensitivity was observed for both AuNSs and HAuNSs at the LSPR IF A at the longer wavelength side than the LSPR peak maximum (B).

**Figure 6:**
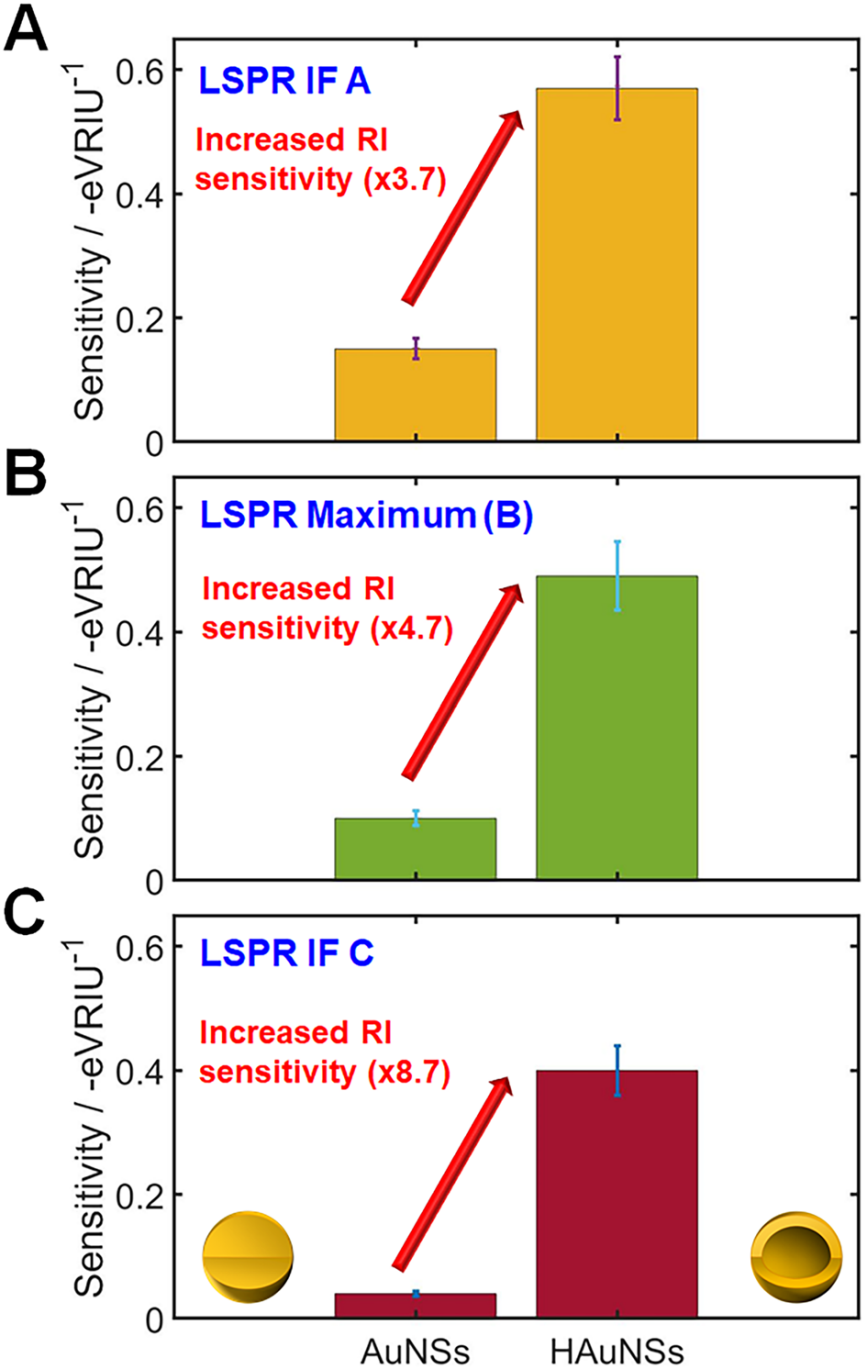
A comparison of RI sensitivity in (**A**) LSPR IF A at the long wavelength side, (**B**) LSPR peak maximum, (**C**) LSPR IF C at the short wavelength side, for solid AuNSs (left) and HAuNSs (right).

We further compared the RI sensitivity at the LSPR maximum and IFs for both AuNSs and HAuNSs. The RI sensitivity of the HAuNSs was 3.7 times better than solid AuNSs at the position of the LPSR IF A (Fig. 6A). The RI sensitivity of the HAuNSs was 4.7 times better than solid AuNSs at the position of the LPSR peak maximum (Fig. 6B). More interestingly, the RI sensitivity of the HAuNSs was 8.7 times better than solid AuNSs at the position of the LPSR IF C at the long wavelength side (Fig. 6C). Thus, the inner cavity of HAuNSs provided a remarkable improvement in the LSPR sensitivity at the LSPR peak maximum and LSPR IFs of HAuNSs. The increased RI sensitivity of HAuNSs having the inner cavity can be mainly ascribed to plasmonic coupling between the inner and outer surface of the Au nanoshell, as depicted in Fig. 3D. The occurrence of plasmonic coupling between the inner and outer surface of the Au nanoshell can lead to the shift of LSPR peak to longer wavelengths as well as the increase of its LSPR sensitivity^19,20^.

**Figure 7:**
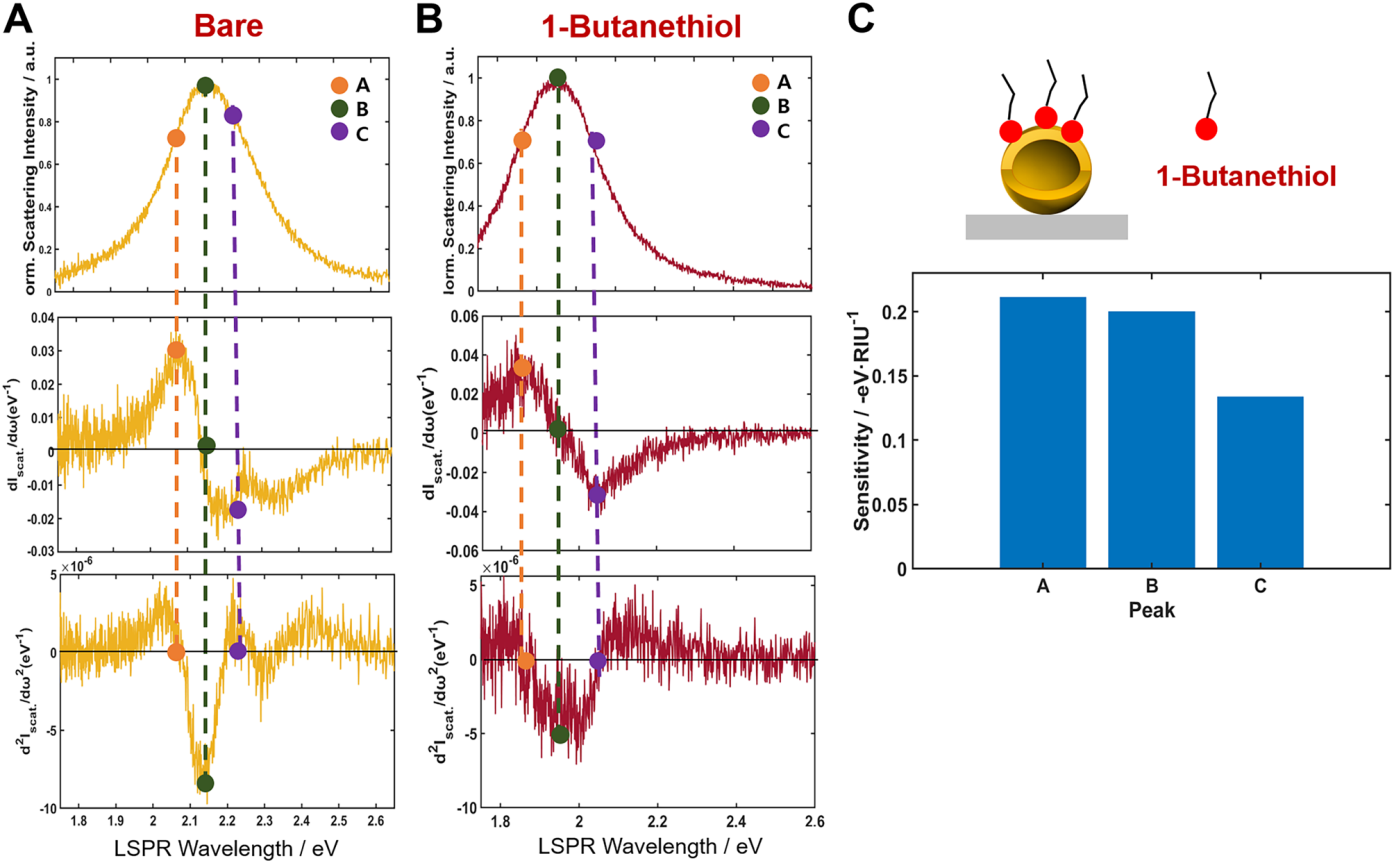
Inflection point method for the single-particle LSPR scattering sensing with HAuNSs in the presence of 1-butanethiol. (**A**, **B**) LSPR scattering efficiencies (first row) and their first (second row) and second (third row) order derivatives. (**C**) Sensitivities of peak shifts in A, B and C induced by the chemisorption of 1-butanethiol.

Finally, thiol reactions with Au have been commonly used in surface modification of Au nanoparticles in LSPR biosensing^21,22^. Thus, we investigated the LSPR peak shifts induced by chemisorption of 1-butanethiol in ethanol on the HAuNSs (Fig. 7 and Fig. S8). As shown in Fig. 7 and Fig. S8, the chemisorption of 1-butanethiol resulted in a redshift in the LSPR scattering spectra of single HAuNSs. Furthermore, the LSPR IF A, located at the long-wavelength side of the LSPR scattering peak, again showed highest sensitivity than the LSPR peak maximum (B) for 1-butanethiol detection (Fig. 7).

## Conclusions

We presented the LSPR sensitivities of single HAuNS with thin Au shell and inner cavity and solid AuNS of similar sizes in three media of air, water and oil possessing different RI values. Higher RI sensitivity was observed at the long wavelength side of the LSPR IF A, than the variation in the frequency of counterpart LSPR maximum peak (B) for both single AuNSs and HAuNSs with a single resonant mode. Furthermore, the single HAuNS showed much higher RI sensitivity at homogeneous LSPR peak maximum and IFs than the single solid AuNS of similar size. The increased RI sensitivity of HAuNSs having the inner cavity was mainly attributed to the occurrence of plasmon coupling between the inner and outer surface of the Au nanoshell. Therefore, this study provides new insight into the effect of an inner cavity of HAuNSs on the RI sensitivity at the homogeneous LSPR IFs. Furthermore, tracking the curvature changes in LSPR scattering spectra of single HAuNSs was found to be effective for the improvement of sensitivity in LSPR-based RI sensing.

## Experimental methods

### Materials

Cetyltrimethylammonium bromide (CTAB)-stabilized AuNSs and HAuNSs were purchased from Nanopartz (Loveland, CO, USA). Immersion oil was purchased from Sigma-Aldrich (St. Louis, MO, USA).

### Characterization of HAuNSs and AuNSs

The structural characterization of AuNSs and HAuNSs was conducted by transmission electron microscopy (TEM, JEL-2100F, JEOL, Japan) to assess the shapes and sizes. Furthermore, the LSPR extinction spectra of the AuNSs and HAuNSs in water were obtained using a Varian Carry 300 UV–Vis spectrophotometer (Agilent, USA).

### Sample preparation for single-particle study

The sample preparation is as following. First, the colloid solution was diluted with distilled water to lower the concentration. The diluted solution was sonicated for 20 min at room temperature and was then dropped on a pre-cleaned glass and covered with a 22 mm × 22 mm No. 1.5 cover glass (Corning, NY). After placing the cover glass, the aqueous solution on the slide glass was dried to achieve the conditions of air as surrounding medium. To achieve the oil as surrounding medium, the same procedure was followed and then, after drying the aqueous solution, the immersion oil was added. The concentration of Au nanoparticles deposited on the glass slide was adjusted to facilitate the measurement of single particles without inter-particle LSPR coupling.

### Single-particle microscopy and spectroscopy

Scattering-based DF microscopy was conducted using an inverted microscope (ECLIPSE Ti-U, NIKON, Japan). A Nikon Plan Fluor oil iris objective (100×) was used with an adjustable NA (0.5–1.3) and a Nikon DF condenser for DF imaging. We obtained DF scattering images by using an Andor EMCCD camera (iXon Ultra 897, UK). The Image J software was used to analyze the collected DF images. Furthermore, we took single particle spectra of AuNSs and HAuNSs by using an Andor spectrometer (SHAMROCK303i, SR-303I-A, UK) equipped with an Andor CCD camera (Newton DU920P-OE, UK). The scattered light from the AuNSs was collected by an objective lens and sent to the entrance of the spectrometer for taking a spectrum. We then dispersed the scattered light by a grating (300 l/mm) inside the spectrometer and detected by the Andor CCD camera (Newton DU920P-OE, UK). A background spectrum was obtained at an area without nanoparticles. Finally, we used Matlab programs specially designed for this study to conduct data analysis.

## Supplementary Information

Supplementary Figures.

## Data Availability

The datasets used during this study available from the corresponding author on reasonable request.
