# Supplementary material for: Enhanced refractive index sensitivity of localized surface plasmon resonance inflection points in single hollow gold nanospheres with inner cavity
Source: Sci Rep. 2022 Apr 28;12:6983. doi: 10.1038/s41598-022-11197-6 (PMC9050728; doi:10.1038/s41598-022-11197-6)
Supplement: Supplementary file 1 — Supplementary Figures. [file 41598_2022_11197_MOESM1_ESM.doc]

<Research Article>

**Supplementary Information**

**Enhanced Refractive Index Sensitivity of Localized Surface Plasmon Resonance Inflection Points in Single Hollow Gold Nanospheres with Inner Cavity**

Yun A Hong^1^ and Ji Won Ha^1,2*^

^1^Department of Chemistry, University of Ulsan, 93 Daehak-ro, Nam-gu, Ulsan 44610, South Korea

^2^Energy Harvest-Storage Research Center (EHSRC), University of Ulsan, 93 Daehak-ro, Nam-gu, Ulsan, South Korea

^*^To whom correspondence should be addressed.

**J. W. Ha**

Phone: +82-52-712-8012

Fax: +82-52-712-8002

E-mail: jwha77@ulsan.ac.kr

**Supplementary Figures**


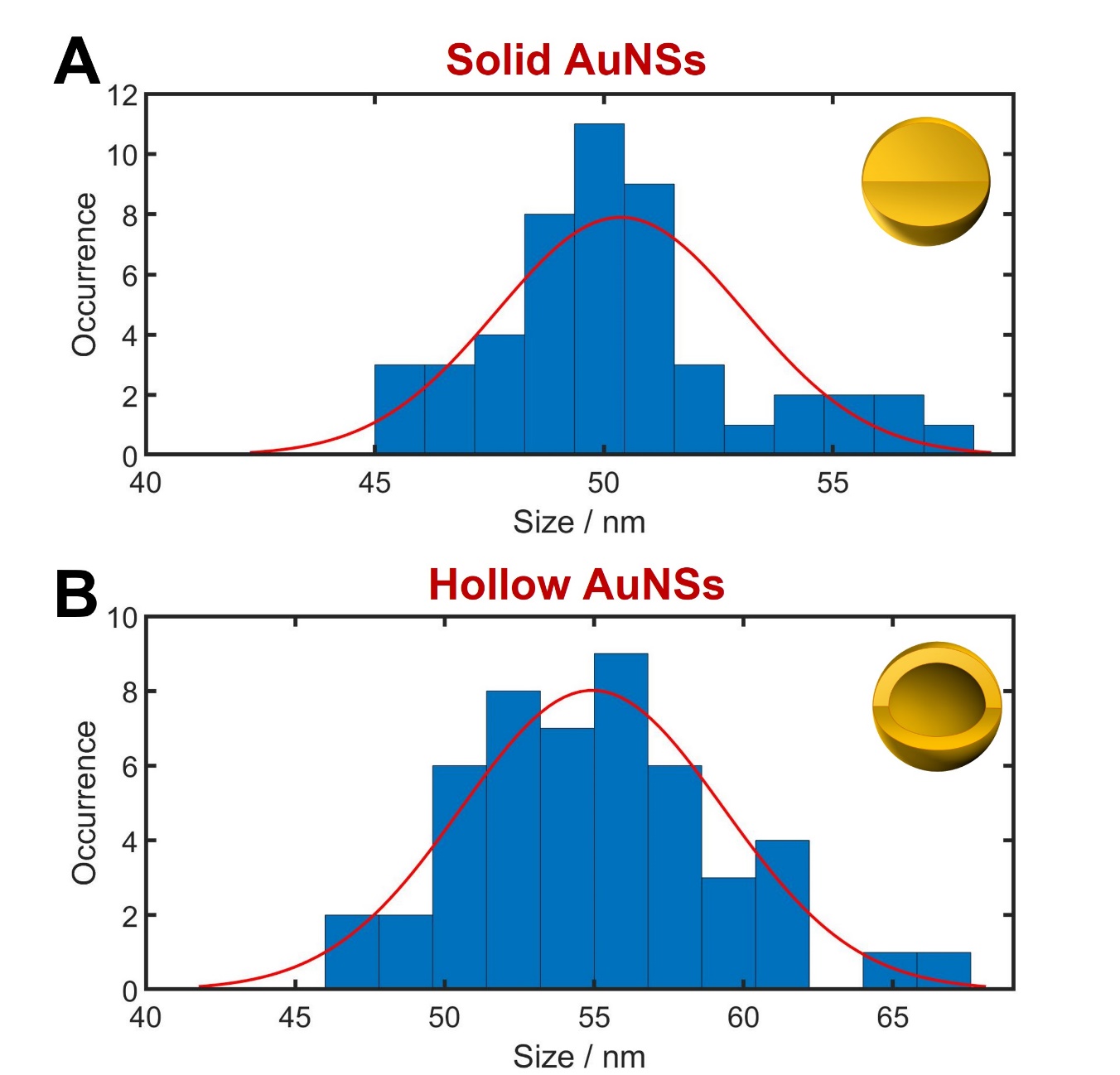


**Fig. S1** **(A)** Histogram to show the size distribution of solid AuNSs. **(B)** Histogram to show the size distribution of HAuNSs with inner cavity.


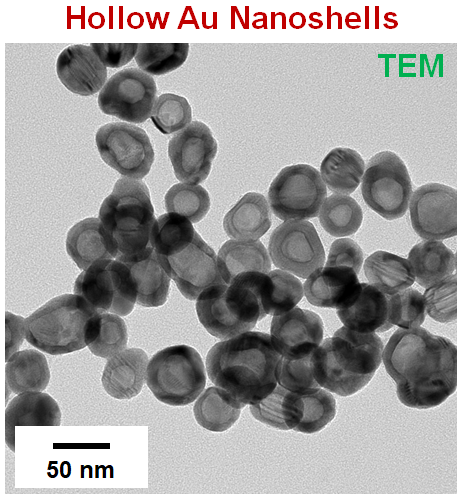


**Fig. S2** TEM image to show multiple HAuNSs used in this study.


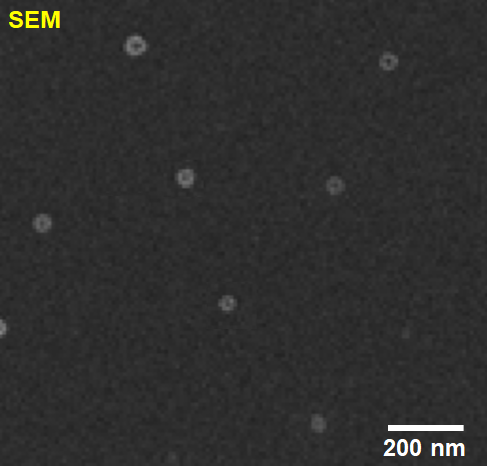


**Fig. S3** SEM image to show multiple HAuNSs used in this study.


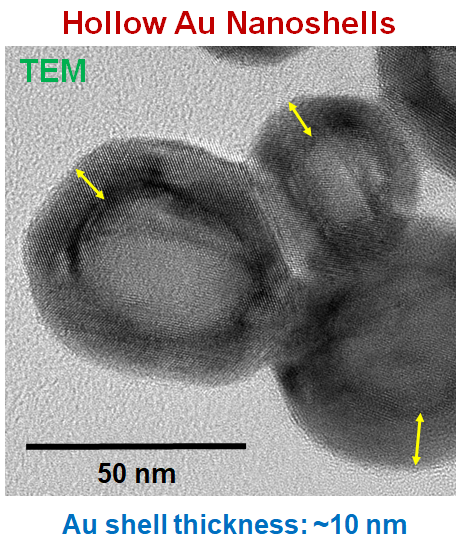


**Fig. S4** TEM image to show the thickness of Au shell in single HAuNSs. The thickness was approximately 10 nm.


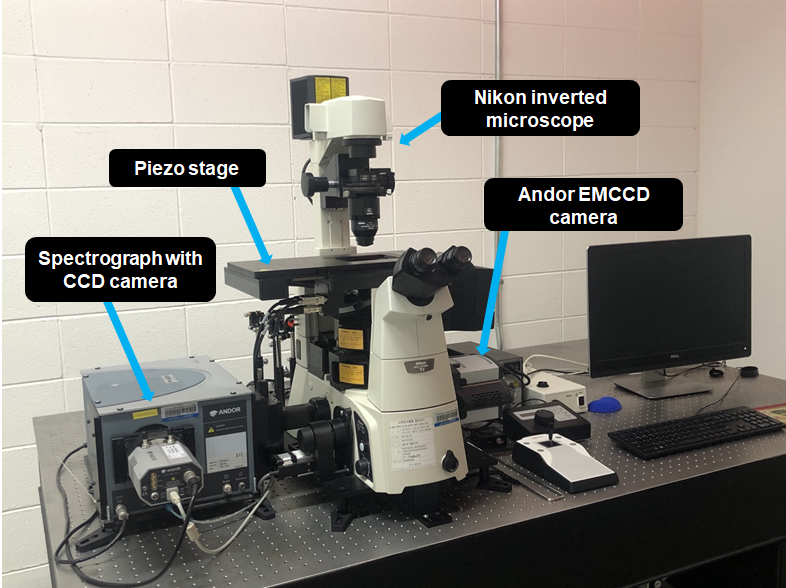


**Fig. S5** A photograph to show the experimental setup for single particle microscopy and spectroscopy.


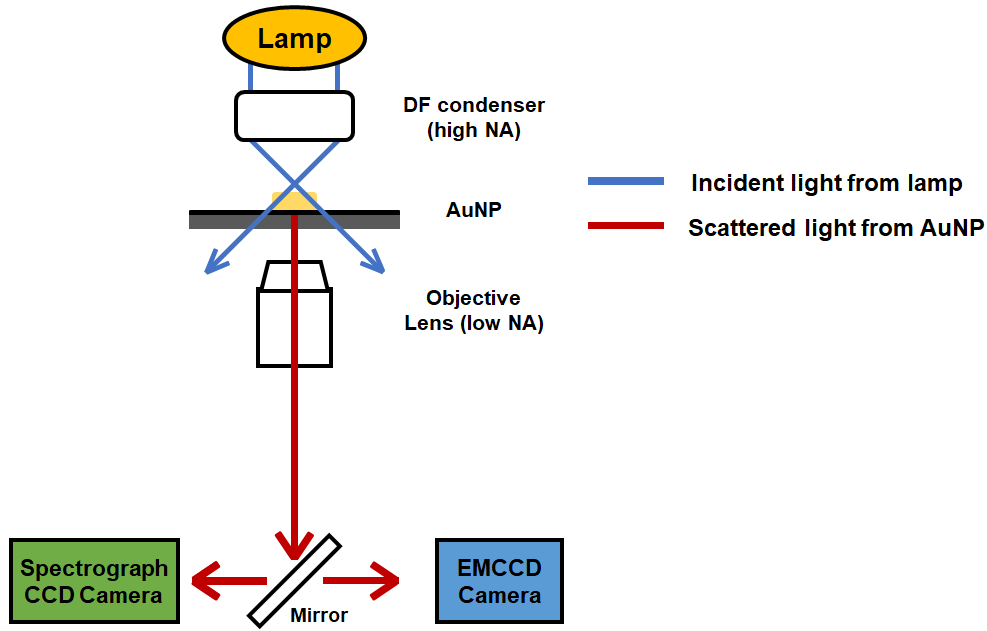


**Fig. S6** Schematic depicting the working principle of scattering-based DF microscopy and spectroscopy.


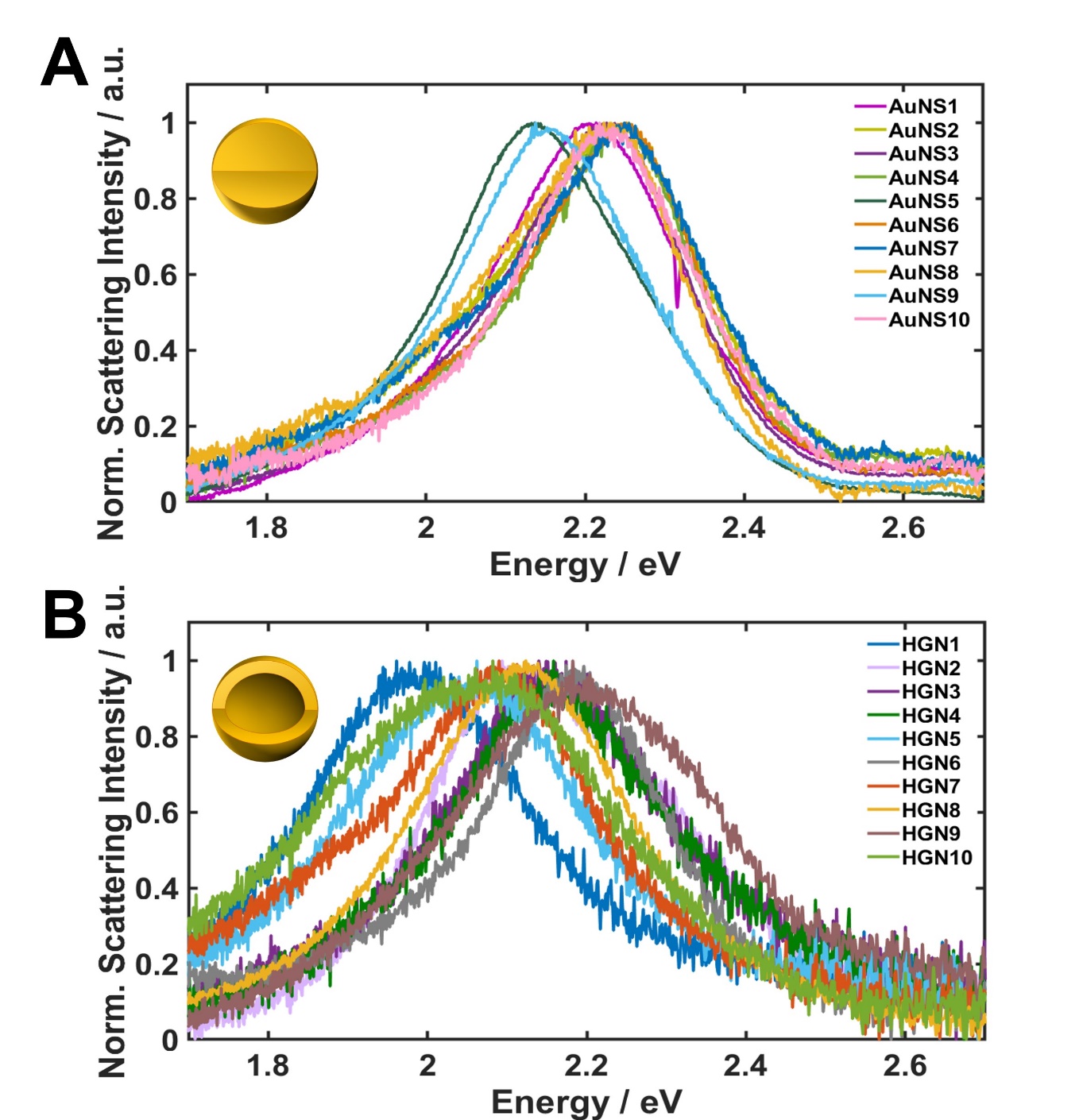


**Fig. S7 (A)** Single-particle scattering spectra of 10 AuNSs. **(B)** Single-particle scattering spectra of 10 HAuNSs.


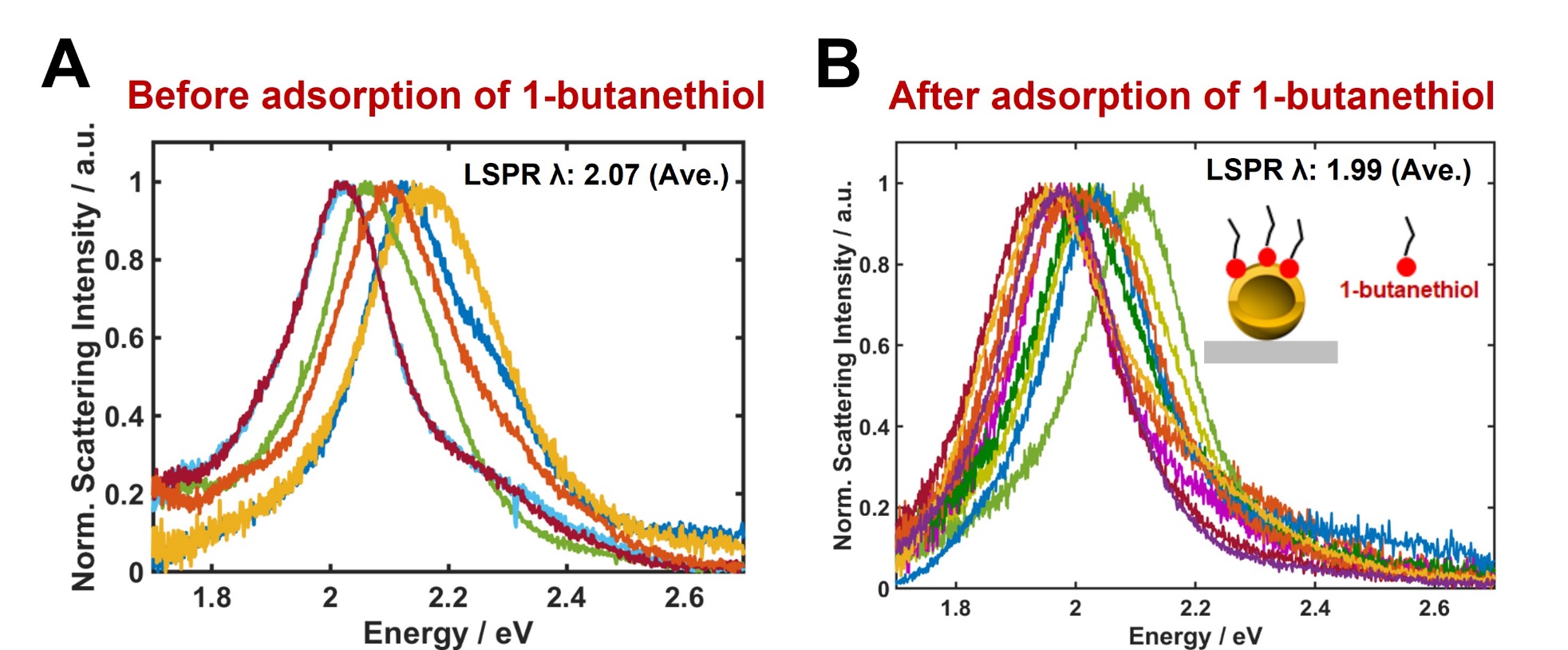


**Fig. S8 (A)** Single-particle scattering spectra of HAuNSs in ethanol before the adsorption of 1-butanethiol. **(B)** Single-particle scattering spectra of HAuNSs after the adsorption of 1-butanethiol in ethanol.
